# A coordinate-based co-localization index to quantify and visualize spatial associations in single-molecule localization microscopy

**DOI:** 10.1038/s41598-022-08746-4

**Published:** 2022-03-18

**Authors:** Jelmer Willems, Harold D. MacGillavry

**Affiliations:** Division of Cell Biology, Neurobiology and Biophysics, Department of Biology, Faculty of Science, Utrecht University, 3584 CH Utrecht, The Netherlands

**Keywords:** Fluorescence imaging, Super-resolution microscopy

## Abstract

Visualizing the subcellular distribution of proteins and determining whether specific proteins co-localize is one of the main strategies in determining the organization and potential interactions of protein complexes in biological samples. The development of super-resolution microscopy techniques such as single-molecule localization microscopy (SMLM) has tremendously increased the ability to resolve protein distribution at nanometer resolution. As super-resolution imaging techniques are becoming instrumental in revealing novel biological insights, new quantitative approaches that exploit the unique nature of SMLM datasets are required. Here, we present a new, local density-based algorithm to quantify co-localization in dual-color SMLM datasets. We show that this method is broadly applicable and only requires molecular coordinates and their localization precision as inputs. Using simulated point patterns, we show that this method robustly measures the co-localization in dual-color SMLM datasets, independent of localization density, but with high sensitivity towards local enrichments. We further validated our method using SMLM imaging of the microtubule network in epithelial cells and used it to study the spatial association between proteins at neuronal synapses. Together, we present a simple and easy-to-use, but powerful method to analyze the spatial association of molecules in dual-color SMLM datasets.

## Introduction

The precise spatial organization of protein complexes within subcellular domains underlies fundamental cellular processes such as migration, cell division and intercellular communication. A central goal in cell biology is therefore to define the mechanisms that control protein distribution and their assembly into functional multi-protein complexes. Fluorescence microscopy evolved to be a powerful and popular strategy to investigate whether specific proteins co-localize and assemble in macromolecular complexes in cells. The development of super-resolution microscopy techniques has tremendously increased the ability to resolve protein distribution at unprecedented resolution^1,2^. In particular, single-molecule localization microscopy (SMLM) techniques such as photoactivated localization microscopy (PALM)^3,4^, (direct) stochastic optical reconstruction microscopy ((d)STORM)^5,6^, point accumulation for imaging in nanoscale topography (PAINT)^7^ and MINFLUX^8^ achieve a spatial resolution down to only a few tens of nanometers. At this scale, protein organization within subcellular compartments and organelles can be effectively investigated and SMLM has been instrumental in numerous recent discoveries in cell biology^9^.

In SMLM, individual fluorophores are stochastically activated so that during image acquisition, sequential frames contain only a small subset of isolated emission events^10,11^. These events are collected over many thousands over frames and can then be localized using computational fitting routines that determine the position of single molecules with nanometer precision. These localizations are then used to reconstruct a super-resolved image with a ~ 10X improved spatial resolution (< 30 nm) compared to conventional, diffraction-limited images. Thus, SMLM images are principally built from a list of molecular coordinates, providing unique opportunities to quantitatively analyze the spatial distribution of molecules in cells and extract new meaningful biological insights^12,13^. As super-resolution imaging techniques continue to undergo significant technical improvements at a rapid pace, there is an increasing demand for quantitative approaches that exploit the unique nature of SMLM datasets.

One approach to analyze SMLM datasets is to use image analysis methods developed for conventional fluorescence images. The coordinate-based data is then first converted to intensity-based, pixelated images by means of binning. As a result, however, considerable information is lost as the gain in resolution by using super-resolution imaging is then partially undone. Also, the conversion to pixelated images relies on additional processing steps that are often parameter-driven, influencing the resulting image. Therefore, a multitude of approaches have been developed that quantify coordinate-based images directly^13,14^. Specifically, statistical spatial analysis methods such as Ripley’s *K* function and derivatives thereof^15,16^, or cross-correlation analysis^17–19^ are often used to determine if observed point patterns are homogeneous, dispersed, or clustered. Furthermore, various dedicated cluster analyses have been developed to detect and segment clusters in point patterns that use hierarchical, density-based clustering algorithms^20,21^, Voronoi tessellation^22,23^, or Bayesian methods^24^.

To explore if proteins interact or assemble in functional complexes in cells, dual-color fluorescence microscopy is often used to detect co-localization of two labeled molecules. Co-localization can then be quantified as the degree of overlap using Manders’ overlap coefficient^25^, or correlation, e.g. Pearson’s *r*^26^ of pixel intensities across the entire image or region of interest (ROI). In SMLM datasets the concept of co-localization however is less trivial to quantify, as two molecules are highly unlikely to be in the exact same position. In SMLM, co-localization is therefore often defined as a measure of intermolecular distance, or spatial association. Several co-localization methods have been developed that directly analyze point patterns by extending spatial analysis methods^27,28^, Voronoi tessellation^29^, or combine cluster detection with co-localization analysis^30–32^. While all these methods have their strengths, they are often embedded in other software pipelines, need user-defined parameters, are focused on detecting co-localization in datasets that contain clusters.

Here, we combined and adapted some of these existing approaches and propose a local density-based co-localization analysis for dual-color SMLM data. We show that it is broadly applicable, does not rely on user-defined setting of parameters and only requires molecular coordinates and their localization precision as inputs. Using computational simulations of SMLM datasets, we show that this method robustly detects spatial association between two channels, independent of localization density. We present this method as a stand-alone MATLAB routine that can be used in combination with virtually all post-imaging processing tools that are used to extract and filter localizations from SMLM images. We further validated our method using SMLM of the microtubule network in U2OS cells and finally applied it to study protein distribution at neuronal synapses. Together, we present a simple and easy-to-use, yet powerful method to measure co-localization in dual-color SMLM datasets.

## Results

### A local density-based co-localization index to quantify spatial association of molecules in dual-color SMLM

In dual-color SMLM experiments, the density of localizations can vary significantly across the field of view and between the two channels. This depends on the local abundance of the labeled proteins, labeling strategy, and data processing and filtering steps. To normalize for these variations, in the first step we calculate the local density (*LD*) value for each localization in a given dataset by counting the number of localizations within a distance *d*. The distance *d* thus determines the sensitivity of the *LD*-value and could be linked to the mean nearest neighbor distance (MNND)^31,33^. However, while theoretically a smaller *d* would be beneficial for the detection of sub-micron differences in localization density, the effective resolution obtained with SMLM data is limited by a combination of both the MNND and the localization error (*ε*)^34,35^. Therefore, the localization error and MNND are determined by taking the average value over all localizations. Together, we use the effective resolution as a single value calculated for the complete dataset as *d.*

$$ d = \sqrt {MNND^{2} + \varepsilon^{2} } $$

In this way, the *LD-value* assigned to each localization represents the density around this localization in a manner that normalizes across variations in density within the full dataset, thus representing the effective resolution.

**Figure 1:**
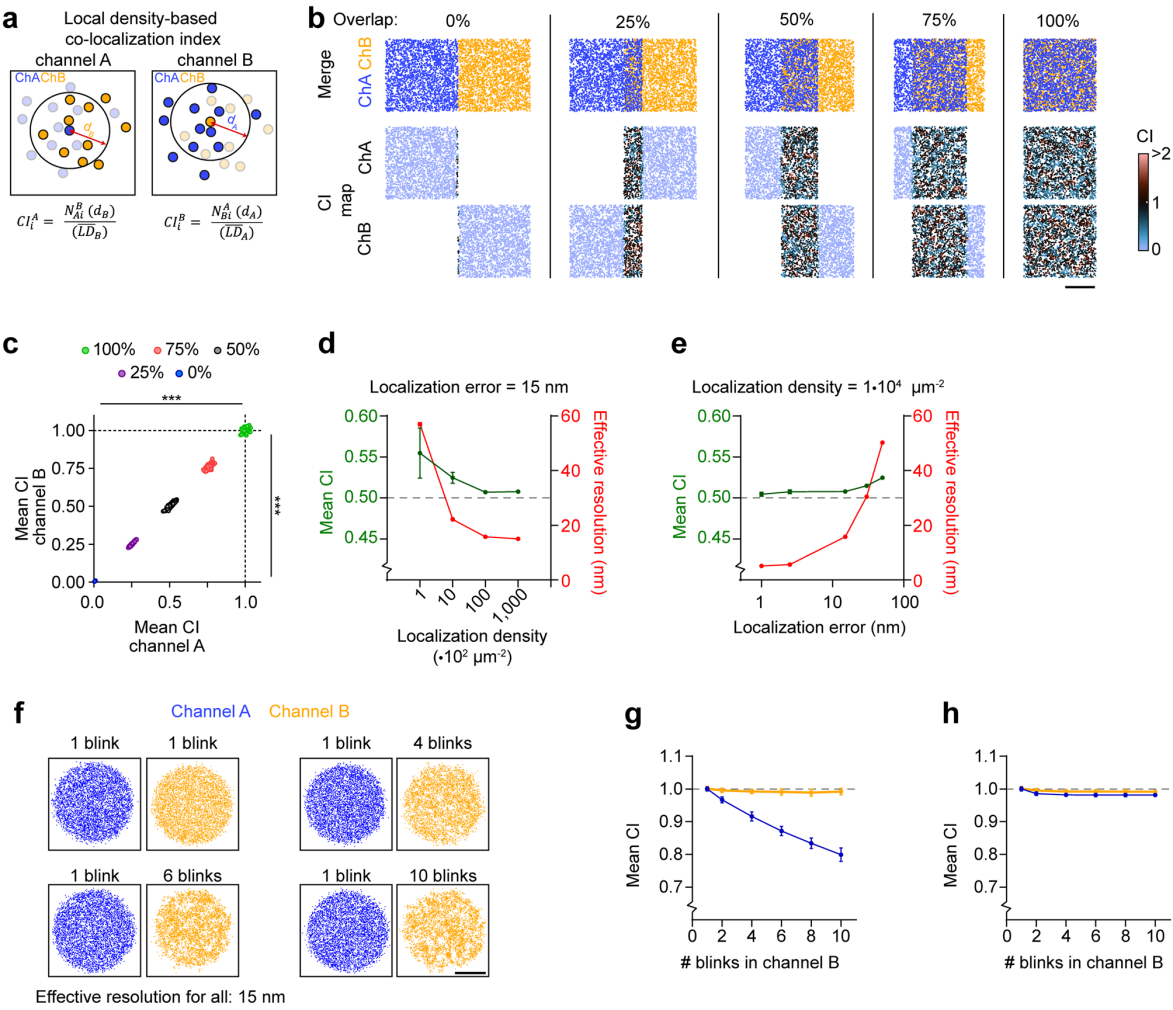
Local density-based co-localization index. (**a**) Concept of coordinate-based co-localization. (**b**) Simulations of point distributions with 1,000 localizations with an average localization error of 15 nm (effective resolution ~ 16 nm) and different amount of overlap between the two channels. Shown are a merge and the co-localization maps. The color code indicates the degree of co-localization. Scale bar, 100 nm. (**c**) Graph of mean co-localization index of 100 simulated point patterns for each of the conditions shown in (**b**). (**d**) Effect of changing localization density on the effective resolution and co-localization outcome (in case of 50% overlapping structures as in A). (**e**) Effect of changing localization precision on the effective resolution and co-localization outcome (in case of 50% overlapping structures as in **a**). (**f**) Simulations of point distributions with increased blinking in channel B (yellow) versus channel A (blue), without changing the total number of localizations. Scale bar, 200 nm. (**g**) Analysis of co-localization for channel A (blue) and B (yellow) related to (**f**). Number of blinks in channel A remained at 1. (**h**) Analysis of co-localization with increased blinking in channel B, thereby also increasing the total number of localizations relative to channel A. Data are represented as means ± SEM. ***P < 0.001, ANOVA. *CI* co-localization index, *LD* local density, *ns* not significant.

Then, to determine the coordinate-based co-localization between two channels, we took a similar strategy as for calculating the *LD-value*. To calculate the co-localization index *CI,* for each localization in channel *A* the number of localizations in channel *B* are counted within the distance set by the effective resolution in channel *B* (Fig. 1a). This number is then divided by the mean *LD-value* of the localizations in channel *B*. Thus, the co-localization index *CI* for the *i*th localization in channel *A* is computed as:

$$ CI_{i}^{A} = \frac{{N_{Ai}^{B} \left( {d_{B} } \right)}}{{\left( {\overline{LD}_{B} } \right)}} $$

where $N_{Ai}^{B}$ is the number of localizations in channel *B* within the distance *d* around the *i*th localization in channel *A*. Conversely, for each localization in channel *B* the *CI* is calculated as:

$$ CI_{i}^{B} = \frac{{N_{Bi}^{A} \left( {d_{A} } \right)}}{{\left( {\overline{LD}_{A} } \right)}} $$

Thus, the co-localization index of any given localization is a measure of the local density of nearby molecules in the other channel (Fig. 1a). If the local density of *Ai* in channel *B* is the same as the average local density in channel B, $CI_{i}^{A}$ will be equal to 1.

To demonstrate this approach, we used simulated dual-color SMLM datasets of randomly distributed localizations with varying degrees of overlap between the channels (ranging from 0 to 100% overlap; Fig. 1b). Since the co-localization index is determined for each localization individually, co-localization can be visualized as a scatter plot color-coded for the co-localization index. These ‘co-localization maps’ visualize the spatial distribution of co-localization between the channels (Fig. 1c). Averaging the co-localization index of these localizations for 100 simulated ROIs showed that, the local density-based co-localization index scales linearly between 0 and 1 for homogenously distributed point patterns, where 0 indicates no co-localization and 1 indicates complete co-localization (co-localization index channel A, 0% overlap: 0.0067 ± 0.0002, 25% overlap: 0.25 ± 0.001, 50% overlap: 0.51 ± 0.002, 75% overlap: 0.76 ± 0.002, 100% overlap: 1.00 ± 0.001, *P* < 0.001, ANOVA, n = 100 point patterns/condition; Fig. 1c). In addition, we performed similar simulations, using sets of multiple overlapping and non-overlapping clusters (Fig. S1a–c) and challenged the method with simulations in which the localizations in channel A are surrounding, but not overlapping localizations in channel B, in a relative confined area (Fig. S2).

Next, we aimed to investigate the effect of variations that usually occur in an experimental setting. First, to investigate the effect of variations in the effective resolution on the observed co-localization, we considered the 50% overlap condition in (Fig. 1b) and varied the localization densities (which influences the MNND) (Fig. 1d) or localization error (Fig. 1e). Importantly, the co-localization index remained close to the ground truth across a wide range of localization densities and localization errors (Fig. 1d,e). The co-localization was only slightly overestimated when the localization error was too high (> 50 nm) or the localization density was too low (< 1000 µm^-2^), i.e., conditions that are generally considered to be insufficient to reconstruct valuable SMLM images. To illustrate this further, we additionally used simulated sets of multiple clusters containing of which half of them have overlap with the second channel and decreased the density of localizations in the second channel (Fig. S1d–f). As expected, co-localization is robustly measured over a large range of densities.

Second, we tested the influence of differences in localization blinking kinetics as organic dyes can undergo multiple on–off switching cycles. To test this, we simulated a spatial pattern without blinking (channel A) and measured overlap with a channel with increasing blinking (channel B) but keeping the total number of localizations between the channels the same (Fig. 1f). With increased blinking, small artificial clusters occurred in channel B, lowering the measured co-localization between the channels, as the spatial distribution of the localizations starts to differ (Fig. 1g). This effect is limited in cases where the labeling density, and thus the number of dyes in the sample, is the same between the channels. As such, more blinking results in more localizations, thereby only marginally affecting the spatial distribution, and thus not influencing the co-localization between the channels (Fig. 1h).

Lastly, we tested the effect of background localizations on the outcome of the co-localization. As all localizations were included in the analysis, increasing the background influenced co-localization outcome (Fig. S1g-–i). Thus, depending on the biological question, background should be taken into account during pre-processing steps prior to co-localization analysis.

In summary, our approach provides a robust method to determine the spatial co-localization between localizations in dual-color SMLM experiments. It provides a measure for the similarity in spatial distribution, independent from density and reflecting the effective resolution. The algorithm is available as a MATLAB function that requires the x–y coordinates of the localizations in the two channels and their localization error as the only inputs (MATLAB Code S1, Method section, and Supplementary test data sets).

### Detection of spatial association within highly crowded regions

In biological samples, proteins are usually not homogenously distributed, but are often concentrated in spatially organized subcellular compartments that contain local variations in enrichments, or nanoscale subdomains^36–38^. Within SMLM datasets, this results in substantial variations in local densities across the analyzed image. We reasoned that our approach would be resistant to this and could reveal local, spatially defined regions of co-localization between two channels even within highly crowded molecular structures. Using simulated point distributions, we tested the influence of differences in local density between channels and the effect of local enrichments on the outcome of the co-localization index in three ways.

**Figure 2:**
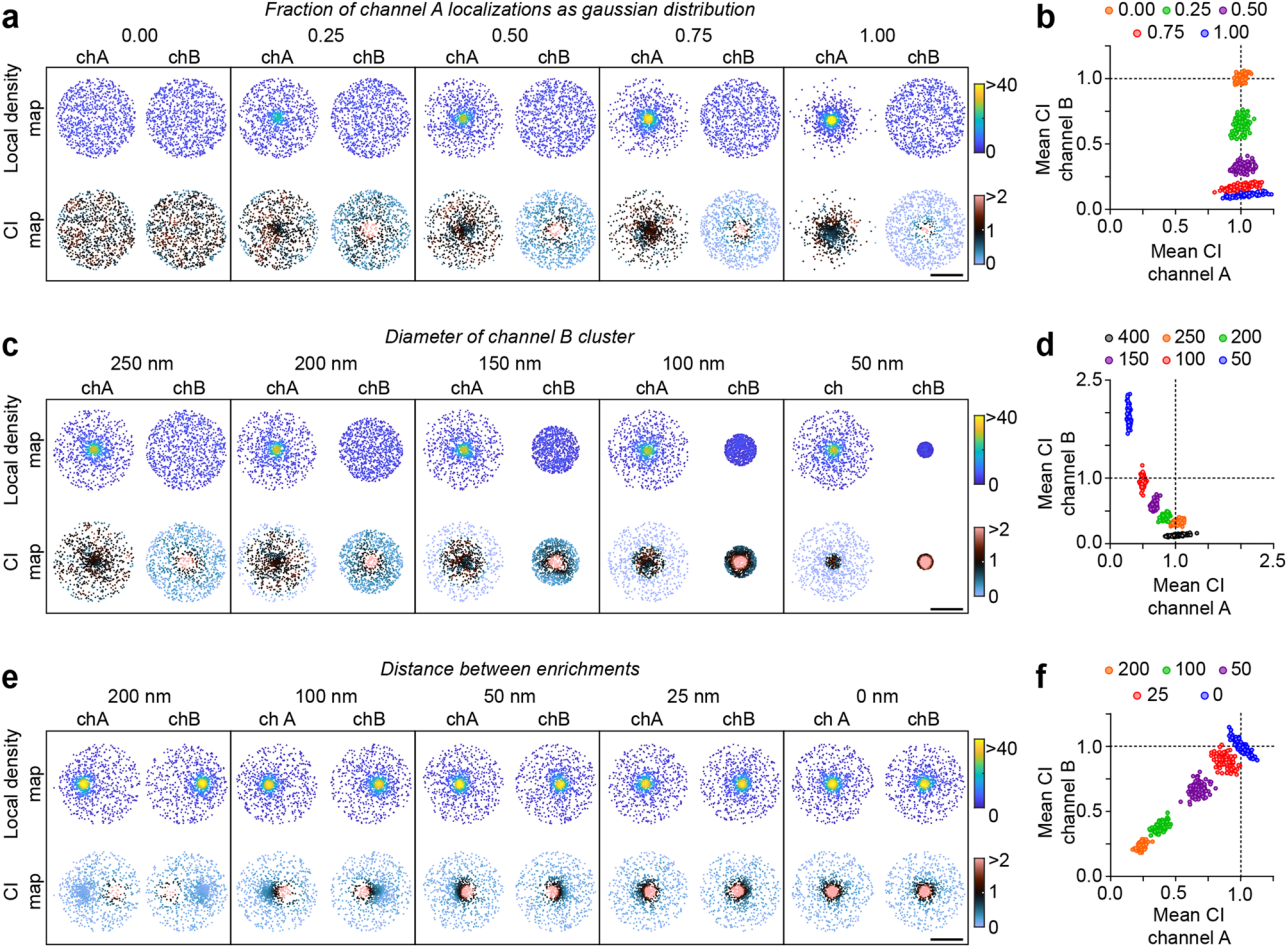
Detection of spatial association within highly confined regions. (**a**) Examples of simulated clusters in which points in channel A are increasingly concentrated towards the center of the cluster whereas channel B remains homogenous in each condition. (**c**) Examples of simulated point patterns with the cluster in channel B varying in size. (**e**) Examples of simulated point patterns in which both channels have a local enrichment with varying distances between the two enrichments. For each of the tests in (**a**, **c**, **e**) a map is shown color-coded for the local density (top) or the co-localization index (CI; bottom). For all clusters, the effective resolution used as search radius was ~ 16 nm. (**b**, **d**, **f**) Graphs showing mean co-localization index for 100 simulated point patterns per condition for the experiments show in (**a**, **c**, **e**) respectively. Scale bar for (**a**, **c**, **e**), 100 nm. *CI* co-localization index, *Ch* channel.

First, we simulated circular clusters with randomly distributed localizations in two channels and varied the local enrichment of localizations in channel A with increasing local density towards the center of the cluster (Fig. 2a). Since the overall distribution in channel B remained similar, the measured co-localization index for channel A remained constant at values around 1 (Fig. 2a,b). In contrast, the co-localization index values of channel B decreased with increasing local densities in channel A (Fig. 2b), as an increasing fraction of localizations in channel B is associated with a relatively lower local density in channel A. Note that the co-localization maps also directly visualize the spatial distribution of co-localization, with lower co-localization index values for channel B at the edges of the cluster where molecules in channel A are less concentrated.

Second, we simulated a scenario where two proteins co-cluster within a structure. To do so, we simulated ROIs in which the localizations in channel B are now also increasingly concentrated, overlapping with the area of high local density in channel A (Fig. 2c). As expected, in this situation the co-localization index values of channel A decrease, as a decreasing fraction of localizations overlap with the localizations in channel B (Fig. 2c,d). For channel B however, the average co-localization index reached values above 1 because these localizations associated with significantly higher (~ 2 ×) local densities in channel A compared to the mean local density of channel A (Fig. 2d). Thus, co-localization values > 1 indicate that the localizations of channel B are enriched towards a local enrichment in channel A. This shows that the co-localization index reliably reports and visualizes co-clustering of localizations that are spatially confined in small structures or domains.

Third, we evaluated the behavior of the co-localization index in simulations of two overlapping clusters, but with non-overlapping local enrichments (Fig. 2e). As expected, the closer these local enrichments were positioned towards each other, the closer the average co-localization index approached a value of 1 (Fig. 2f). Note that the average co-localization index is close to 1 when two random distributions overlap (Fig. 2a, first example), but also when local enrichments within clusters completely overlap (Fig. 2e, last example) as in both cases the localizations in the two channels have the same distribution. Thus, co-localization as measured here reports the similarity in spatial distribution. Nevertheless, the spatial distribution of the co-localization values of the individual localizations visualized in the co-localization maps revealed clear differences in the extent and position of co-localization between the two conditions. As mentioned earlier, the degree by which co-localization can be detected between two objects (or two objects can be considered non-overlapping), is dependent on the effective resolution of the data^34,35^. To test this further, we repeated the simulations of Fig. 2e, varying the localization error (Fig. S3). As expected, higher effective resolution, i.e., lower localization error, allows for higher sensitivity towards detecting partially overlapping structures.

Together, these simulations demonstrate that our method provides a robust analysis of spatial co-localization between two sets of localizations that is intuitive to interpret and provides a visual output of local co-localization. Our method thus measures co-localization, as a measure of similarity in spatial distributions, independent of density and even revealing co-clustering of proteins within densely crowded structures.

### Detecting co-localization in biological samples

**Figure 3:**
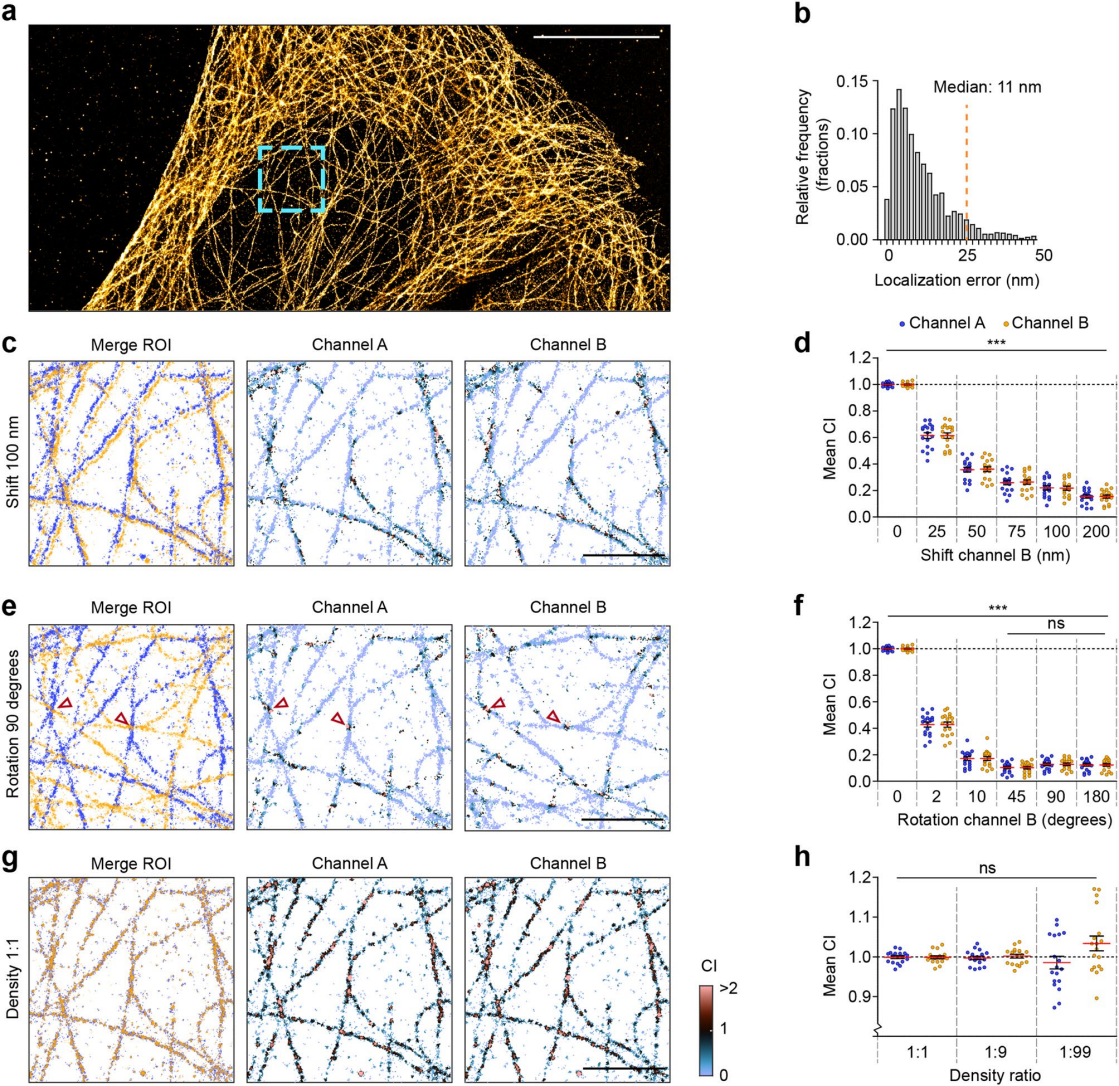
Application and validation of co-localization index on a biological sample. (**a**) dSTORM imaging was performed on U2OS cells labeled with anti-alpha-tubulin antibodies. Shown is a rendered reconstruction of all localizations. Scale bar, 10 μm. (**b**) Frequency distribution of the localization precision across all localizations. Localizations with localization precision > 25 nm (orange line) were removed from the dataset. Bin size, 2 nm. (**c**) Example ROI in which channel B is shifted 100 nm to the right relative to channel A. (**d**) Graph showing mean co-localization with varying amounts of shift. (**e**) Example in which channel B is rotated 90 degrees relative to channel A. Two microtubule cross-points are indicated with red arrows Scalebar in (c, e, g), 2 μm. (**f**) Graph showing mean co-localization for individual ROIs with varying degree of rotation of channel B. (**g**) The localization dataset was randomly split into two channels (A, blue; B, yellow), with varying density ratios. Shown is an example ROI, indicated with a blue box in (a) with a 1:1 ratio. Also shown are the maps color-coded for the co-localization index. (**h**) Graph showing mean co-localization index for individual ROIs with varying density ratios. N = 18 ROIs from 3 cells. Data are represented as means ± SEM. Ns, not significant, ***P < 0.001, ANOVA. *ROI* region of interest, *CI* co-localization index.

Next, we wished to validate the co-localization index on experimental data from well-characterized cellular structures. We therefore labeled the microtubule network in U2OS cells using immunostaining for alpha-tubulin and acquired SMLM images using dSTORM (Fig. 3a). Localizations with a localization precision < 25 nm (Fig. 3b) were selected for rendering a super-resolution image. Then, to approach perfect co-localization, we randomly divided all localizations into two channels (1:1) and determined the co-localization across multiple ROIs. As expected, the individual channels revealed close co-localization between the two channels (Fig. 3c), resulting in average co-localization values very close to 1 (co-localization index: 1.00 ± 0.003 for channel A and 1.00 ± 0.004 for channel B, n = 18 ROIs from 3 cells; Fig. 3c,d). To test the sensitivity of the co-localization index, we tested conditions in which the localizations of channel B were laterally shifted or rotated relative to channel A. The introduction of small amounts of lateral shift significantly lowered the co-localization within ROIs (25 nm: 0.61 ± 0.02, 50 nm: 0.36 ± 0.02, 75 nm: 0.26 ± 0.02, 100 nm: 0.22 ± 0.02, 200 nm: 0.15 ± 0.01, *P* < 0.001, ANOVA; Fig. 3c,d). Similarly, rotating channel B relative to channel A significantly reduced the co-localization (2 degrees: 0.42 ± 0.02, 10 degrees: 0.17 ± 0.01, 45 degrees: 0.10 ± 0.009, 90 degrees: 0.12 ± 0.009, *P* < 0.001, ANOVA; Fig. 3e,f). Note that the residual co-localization is mostly the result of remaining microtubule cross-points (Fig. 3e), indicating the sensitivity of this approach and the usefulness of visualizing the co-localization maps.

Next, we randomly split the same dataset with varying relative molecular densities (1:9 and 1:99) between the channels. As expected from the simulations in Fig. 1 and S1, changing the localization density did not significantly alter the outcome of the co-localization analysis (density ratio 1:9, 1.00 ± 0.004 for channel A and 0.99 ± 0.005 for channel B, density ratio 1:99, 0.99 ± 0.01 for channel A and 1.03 ± 0.01 for channel B, *P* > 0.05, ANOVA; Fig. 3g,h).

We next used these datasets to compare our method to two existing co-localization methods (Fig. S4). First, we compared our method with ClusDoC^32^. ClusDoc uses an optimized cutoff of 0.4 on the -1 (anti-correlated) to + 1 (perfectly correlated distributions) scale developed earlier by Malkusch et al^28^, to obtain a value for the percentage of co-localizing localizations. Our approach (Fig. S4a) outperformed ClusDoC, as the latter showed a higher sensitivity to differences in density between the two channels (Fig. S4b). Second, we tested Coloc-Tesseler, which uses Voronoi diagrams with Manders’ and Spearman correlations as readouts^29^. Similar to our approach, the Manders’ correlation readout of Coloc-Tesseler correctly measured the co-localization in two overlapping channels with similar distribution and densities, although slightly underestimating co-localization when the density in one of the channels becomes lower (Fig. S4c). In contrast, we found the Spearman correlation to consistently underestimate co-localization, similar as shown earlier^29^, and more sensitive to changes in density between the channels (Fig. S4d).

Together, these results demonstrate that the local density-based co-localization index presented here is a simple, yet very powerful tool in detecting and visualizing the degree of spatial co-localization in biological samples.

### Detecting co-localization in dual-color SMLM images of neuronal synapses

As a final test case of our method, we turned to neuronal synapses. At synapses, a plethora of protein species are closely packed, both at the presynaptic active zone that is closely aligned with postsynaptic proteins, with only several nanometers between them. The subsynaptic clustering and relative positioning of synaptic proteins is critically important for synaptic transmission^31,33,39,40^.

**Figure 4:**
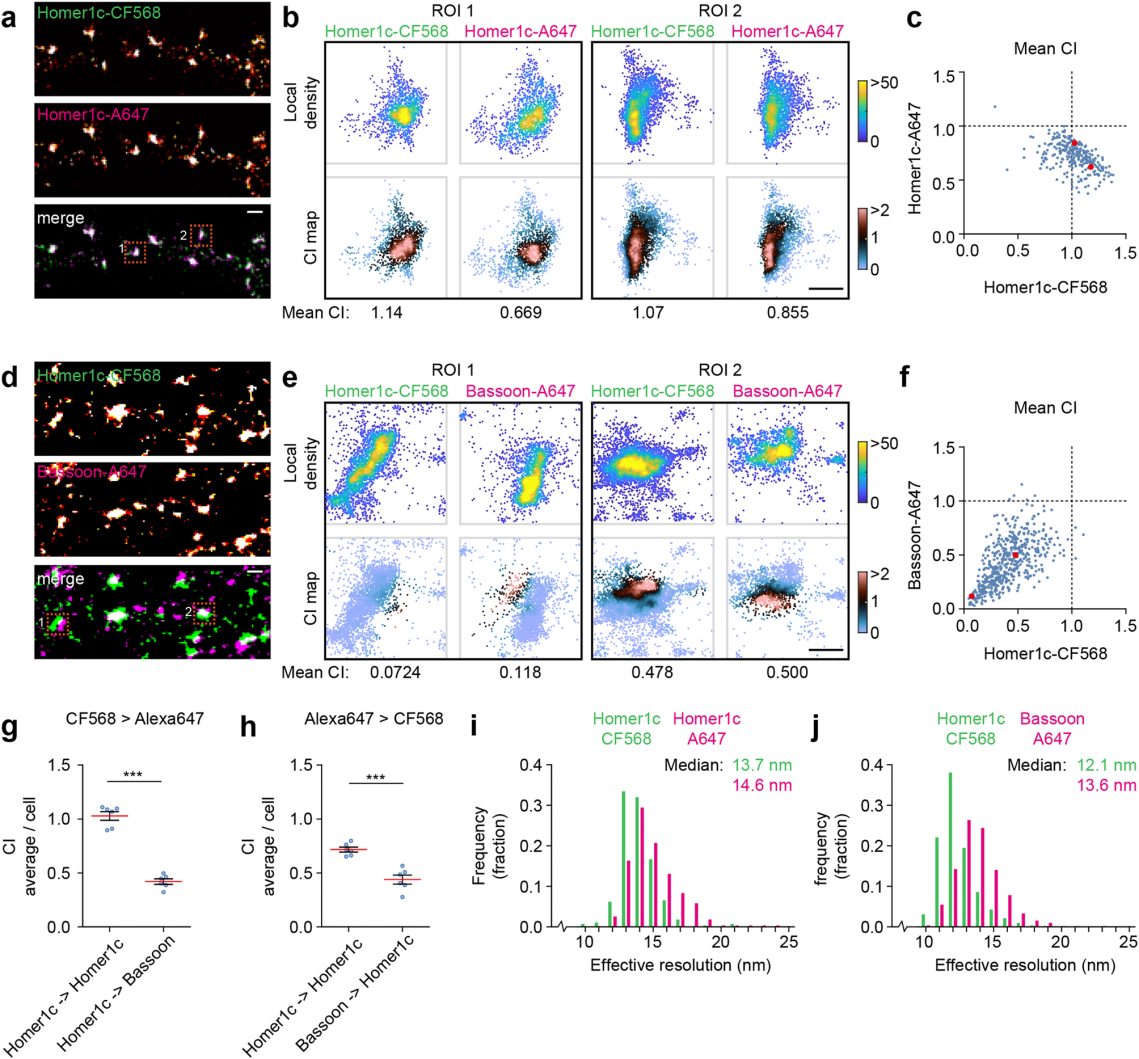
Measuring co-localization at neuronal synapses. (**a**) Rendered image of dual-color dSTORM on Homer1c in the dendrite of a hippocampal neuron. Homer1c was labeled with both CF568 (green) and Alexa647 (magenta). Scale bar, 500 nm. (**b**) Plots of two example ROIs (indicated with orange boxes in A). Shown are plots color-coded for the local density as well as the co-localization index. Scale bar, 200 nm. (**c**) Graph of mean co-localization index for 282 synapses from 6 neurons. Example synapses shown in B are plotted in red. (**d**) Rendered image of dual-color dSTORM on Homer1c labeled with CF568 (green) and Bassoon labeled with Alexa647 (magenta). (**e**) Example plots of ROIs indicated with orange boxes in (**d**). Scale bar, 200 nm. (**f**) Graph of mean co-localization index for 304 synapses from 6 neurons. Example synapses shown in E are plotted in red. (**g**, **h**) Graphs showing mean co-localization index averaged per neuron for the CF568 channel (**g**) and Alexa647 channel (**h**), comparing the co-localization of Homer1c and Bassoon. (**i**, **j**) Frequency distribution plots showing the calculated effective resolution and search radius *d*. Bin size, 1 nm. Data are represented as means ± SEM. ***P < 0.001, Student t test. *ROI* region of interest, *CI* co-localization index.

We reasoned that our co-localization index might be very well-suited for measuring the degree of spatial association between proteins in these dense structures. To this end, we performed dual-color dSTORM on cultured hippocampal neurons co-labeled for the postsynaptic protein Homer1c and the presynaptic protein Bassoon. We first labeled the postsynaptic protein Homer1c with both CF568- and Alexa647- (A647) coupled secondary antibodies (Fig. 4a). As expected, the labeling in the two channels overlapped almost completely and we found a high degree of co-localization between the two channels, also apparent from the co-localization maps of individual synapses (Fig. 4b,c). Next, we analyzed the co-localization index between Homer1c and Bassoon localizations (Fig. 4d,e). As expected, the co-localization between these proteins was significantly lower, and varied largely between individual ROIs, indicative for only partial overlap (co-localization index CF568 > Alexa647: 1.03 ± 0.04 for Homer1c > Homer1c and 0.42 ± 0.03 for Homer1c > Bassoon, n = 6 neurons, *P* < 0.001, Student *t* test; Fig. 4g), (co-localization index Alexa647 > CF568: 0.72 ± 0.023 for Homer1c > Homer1c and 0.44 ± 0.04 for Homer1c > Bassoon, n = 6 neurons, *P* < 0.001, Student *t* test; Fig. 4h). Importantly, the average co-localization values corresponded well with the observed overlap: synapses with non-overlapping Homer1c-Bassoon clusters had a low average co-localization index while synapses with partially overlapping clusters resulted in a higher average co-localization index (example synapses, Fig. 4e). Note that variations in co-localization index most likely result from the relative orientations of presynaptic and postsynaptic structures with respect to the focal plane (Fig. 4f). Despite differences in the biophysical properties of these dyes which are partially reflected by small differences in the observed effective resolution (Fig. 4i,j), our approach robustly reports co-localization at sub-diffraction resolution. Together, these results show that the co-localization index as described here, can be used as a reliable method to quantify the spatial association of molecules in dual-color SMLM data.

## Discussion

The development of SMLM approaches has revolutionized cell biology permitting the precise investigation of the nanoscale organization of protein within cells. SMLM images are essentially coordinate-based datasets, providing unique opportunities to quantify the spatial distribution of molecules. Here, we present a method to quantify the spatial association of molecules in dual-color SMLM data. We first benchmarked the method in silico with various simulated point patterns that mimic commonly found distributions in biological samples. We found that our method is resistant to variations in overall localization density and adequately detects spatial co-localization in fields with randomly distributed points, as well as within highly crowded clusters. Importantly, our method takes the localization density and localization error into account, such that the reported co-localization is on the scale relative to the effective resolution^34,35^ of the dataset. We noted from simulated as well as experimental data, that the accuracy of the localization index is more sensitive to the localization density than the localization precision. Ideally, the effective resolution is thus limited by the localization precision and not the molecular density. In addition, we tested the effect of blinking kinetics and background localizations on the outcome of co-localization. We furthermore showed that our co-localization method is versatile and can be applied to various types of biological samples, including the microtubule network in epithelial cells, a sample with distinct differences in localization density across the field of view, and neuronal synapses, structures with a highly dense accumulation of heterogeneously distributed proteins.

We also compared our co-localization index with previously described methods, Coloc-Tesseler^23^ and ClusDoC^32^. While Coloc-Tesseler performed well when using the Manders’ correlation as an output, ClusDoC underestimated co-localization in datasets with varying densities. Of note, the definition of co-localization in ClusDoC (which integrates a co-localization method developed by Malkusch et al.^28^), is different from the method presented here, as ClusDoC measures co-clustering. Co-localization as measured in our method, provides a measure for the similarity in spatial distributions between the channels, and is thus independent of localization density, and does not rely on (co-)clustering of molecules.

In conclusion, the method presented here provides a versatile approach to quantify co-localization in dual-color SMLM experiments. The algorithm is available as a MATLAB function, which can be easily incorporated into existing analysis pipelines and could potentially be extended to 3D datasets and triple- or more -channel experiments. Thus, we believe that the co-localization method described here, will serve as a fast and easy-to-use approach to measure spatial associations between molecules in multi-color SMLM-datasets and will be instrumental in unveiling the molecular organization in subcellular compartments.

## Methods

### Antibodies

Primary antibodies used in this study are the following: Mouse anti-alpha-tubulin ([B-5-1-2], Sigma, T5168, RRID AB_477582), Rabbit Anti-GFP (MBL Sanbio, 598, RRID AB_591819) and mouse anti-Bassoon ([SAP7F407], Enzo, ADI-VAM-PS003-F, RRID AB_10618753). Alexa647-conjugated secondary antibodies were from Life Technologies (A21236 and A21245). CF568 conjugated secondary antibodies were from Sigma (SAB4600085).

### Ethics statement

All experiments were approved by the Dutch Animal Experiments Committee (Dier Experimenten Commissie [DEC], work protocol project number: AVD1080020173404), performed in line with institutional guidelines of Utrecht University, and conducted in agreement with Dutch law (Wet op de Dierproeven, 1996) and European regulations (Directive 2010/63/EU). Timed pregnant Wistar rats were obtained from Janvier Labs. The study was carried out in compliance with the ARRIVE guidelines.

### DNA-plasmids

pCMV-Homer1c-GFP^41^.

### Calculation of local density and co-localization index

The local density is determined for each localization in both channels. First, for each localization, the NND is determined within a given ROI using the MATLAB function *knnsearch*, with input (x,y,’k’,2), and output [IDX,D], to obtain the distance (D) for the first nearest neighbor that is not itself. The NND for each localization was averaged to obtain the MNND. Next, for each localization, the local density (*LD*) is defined as the number of localizations within a radius defined by the effective resolution making use of the MATLAB function *rangesearch*. For each channel, the *LD* values are averaged together to obtain *LD*_*A*_ and *LD*_*B*_*.* Effective resolution was calculated as^34^,

$$ effective\;resolution = \sqrt {MNND^{2} + \varepsilon^{2} } $$

where $\varepsilon$ is the localization error, determined as the average of the complete dataset.

The co-localization index (CI) is determined as the number of localizations of channel B (*N*) within a radius (*d*) around each localization in channel A (*Ai*) normalized to the mean local density of the localizations in channel B ($\overline{LD}_{B}$), with *d* being the effective resolution of the localizations in channel B.

$$ CI_{i}^{A} \left( d \right) = \frac{{N_{Ai}^{B} \left( {d_{B} } \right)}}{{\overline{LD}_{B} }} $$

Thus, for the second channel, the CI values are calculated as:

$$ CI_{i}^{B} \left( d \right) = \frac{{N_{Bi}^{A} \left( {d_{A} } \right)}}{{\overline{LD}_{A} }} $$

The CI values calculated for each localization individually can be used to plot a co-localization map and averaged to obtain a single mean-CI value of the full ROI for both channels.

We integrated these calculations in a MATLAB function (Code S1) with the input being the x and y coordinates of channel A and B and their localization errors. The function will return a MATLAB structure, with fields containing the average co-localization index for each channel, the calculated effective resolution (*d*) as well as the CI values for the individual localizations (See Code S1).

### Simulations

To generate random localization clusters in squares, for each x and y coordinate, a value *ρ* (between 0 and 1) was generated using the MATLAB function *rand*. x and y were calculated as *ρ* multiplied by the cluster dimensions (250 × 250 nm). This was repeated for the second channel. Differences in overlap were generated by increasing the x coordinate of the second channel (+ 62.5 nm for every 25% decrease in overlap). For each condition, we simulated 100 clusters.

For circular clusters, randomly distributed x and y coordinates were calculated as ${\text{x}} = \sqrt {\rho } \cdot \cos \left( \theta \right) \cdot d$ and ${\text{y}} = \sqrt {\rho } *\sin \left( \theta \right)*d$, were *θ* is a random angle (between 0 and 2π) and *d* is the diameter of the full cluster. Background localizations were simulated by generating randomly placed coordinates in a square as described above at a density based on the signal/background ratio of the clusters. Background localizations were then removed from the clusters themselves. Clusters with a local enrichment were generated by combining a set randomly distributed localizations with a set localizations distributed as gaussian distribution. For gaussian distributions, values *ρ* were generated using the MATLAB function *randn*. x and y were calculated as x = *σ ⋅ ρ ⋅* cos(*θ) ⋅ d* + *dc* and y = *σ ⋅ ρ ⋅* sin(*θ) ⋅ d,* with *σ* being the standard deviation (0.33 for the examples in Fig. 2a,c and 0.25 in Figs. 2e and S1), *θ* and *d* as described above, and *dc* being the distance from the center of the cluster (in nm). For the simulations shown in Fig. 2a we combined randomly distributed localizations with localizations in a gaussian distribution in varying ratios (1:0, 3:1, 1:1, 1:3, 0:1). For simulations in Fig. 2e, we used a ratio of 1:1, but changed *dc* to position the local-enrichment relative to the center of the cluster. For all conditions, we simulated 100 independent point patterns.

Localization error was simulated by offsetting the x and y coordinates with random distance, derived from a gaussian distribution (MATLAB function *randn*), with the sigma being the intended localization error. A localization error of 15 nm was used if not indicated differently. Blinking was simulated by adding additional localizations around existing ones, with offset derived from a Gaussian distribution (MATLAB function *randn*), with the sigma varied as the localization error.

### U2OS cell culture

U2OS cells (ATCC HTB-96) were grown in DMEM (Lonza) supplemented with 10% fetal calf serum (Sigma), 2 mM glutamine and 1% penicillin and streptomycin (pen/strep) (Gibco). One day before fixation and immunostaining, cells were plated on 18-mm coverslips and grown to ~ 50% confluency.

### Immunolabeling of microtubules in U2OS cells

U2OS cells were incubated in pre-warmed (37 °C) cytoskeleton extraction buffer (PEM80 buffer [80 mM PIPES, 1 mM EGTA, 2 mM MgCl2 (pH 6.9)], 0.3% [v/v] Triton-X100, 0.1% [v/v] glutaraldehyde) for 1 min at room temperature (RT). Next, the cells were further fixed using 4% (w/v) paraformaldehyde (PFA) and 4% (w/v) sucrose in phosphate-buffered saline (PBS) (PFA/Suc) for 10 min at RT, washed three times for 5 min with PBS containing 0.1 M glycine (PBS/Gly), and subsequently incubated with 1 mg/ml sodium borohydride in PBS for 7 min at RT. Coverslips were washed 3 times for 5 min with PBS/Gly. Next, the coverslips were incubated in 2% bovine serum albumin (BSA) and 0.1% Triton-X100 in PBS/Gly for 1 h at 37 °C. Next, microtubules were labeled using anti-alfa-tubulin primary antibody, diluted 1:1000 in 1% BSA [w/v], 0.1% [v/v] Triton-X100 in PBS/Gly, overnight at 4 °C. Cells were washed three times 5 min with PBS and incubated with goat anti-mouse secondary antibodies conjugated to Alexa647 diluted 1:400 in 1% [w/v] BSA, 0.1% [v/v] Triton-X100 in PBS/Gly. After one hour, cells were washed three times 5 min with PBS and subsequently post-fixed with PFA/Suc for 5 min. Cells were washed three times with PBS/Gly after which cells were kept in PBS till imaging.

### Dissociated neuron cultures

Dissociated hippocampal cultures were prepared from embryonic day 18 (E18) rat brains of both genders, as described in^42^ and in accordance to the approved DEC work-protocol as mentioned in the ethics statements above. Mother rats were sacrificed by gradual fill CO2/O2. Subsequently the uterus containing the pups is taken out and is stored in a sterile ice cold environment. After the pups were sedated by the cold, they were removed from the uterus and decapitated. Dissociated neurons were plated on Ø18-mm coverslips coated with poly-L-lysine (37.5 µg/ml, Sigma-Aldrich) and laminin (1.25 µg/ml, Roche Diagnostics) at a density of 100,000 neurons per well. Neurons were grown in Neurobasal medium (NB) supplemented with 1% pen/strep, 2% [v/v] B27, and 0.5 mM L-glutamine (all from Gibco) (NB-complete medium) at 37 °C in 5% CO_2._ From days in vitro (DIV) 1 onward, medium was refreshed weekly by replacing half of the medium with Brainphys neuronal medium (BP) supplemented with 2% [v/v] NeuroCult SM1 neuronal supplement (STEMCELL Technologies) and 1% pen/strep (BP-complete medium).

### Transfection of hippocampal neurons

Neurons were transfected at DIV 15 using Lipofectamine 2000 reagent (Invitrogen). Briefly, for one coverslip, 1–2 μg DNA was mixed with 3.3 μl Lipofectamine in 200 μl BP medium and incubated for 30 min at RT. Next, 500 μl conditioned medium was transferred to a new culture plate and replaced by 300 μl BP supplemented with 0.5 mM L-glutamine. The DNA mix was added to the neurons and incubated at 37 °C and 5% CO2. After 90–120 min, neurons were transferred to the new culture plate with conditioned medium and 500 μl fresh BP-complete medium and kept at 37 °C and 5% CO_2_ till fixation at DIV 21.

### Immunolabeling of neuronal cultures

Neurons were fixed at DIV 21 in PFA/Suc for 10 min at RT after which they were washed 3 times 5 min in PBS/Gly. Next, the coverslips were incubated in blocking buffer (10% [v/v] normal goat serum [NGS] (Abcam), in PBS/Gly with 0.1% [v/v] Triton X100) for 1 h at 37 °C. Next, coverslips were incubated with primary antibodies diluted in incubation buffer (5% [v/v] NGS in PBS/Gly with 0.1% [v/v] Triton X100) overnight at 4 °C. Coverslips were next washed 3 times 5 min with PSD/Gly and incubated with secondary antibodies diluted 1:400 in incubation buffer for 1 h at RT. Next, coverslips were washed 3 times 5 min in PBS, post fixed in 4% PFA/Suc for 5 min, additionally washed 3 times with PBS/Gly, and kept in PBS at 4 °C until imaging.

### SMLM and filtering of raw localization data

SMLM experiments were performed using the Nanoimager microscope (Oxford Nanoimaging; ONI) equipped with a 100 × oil-immersion objective (Olympus Plan Apo, NA 1.4) and an XYZ closed-loop piezo stage. Imaging was performed using 473-nm, 561-nm and 640-nm lasers for GFP, CF568 and Alexa647 excitation respectively. Fluorescence was detected using a sCMOS camera (ORCA Flash 4, Hamamatsu). Integrated filters were used to split far-red emission onto the right side of the camera and blue-green–red emission spectra on the left side, enabling simultaneous dual-color imaging.

The imaging chamber was temperature-controlled at 30 °C to prevent fluctuations in temperature during the time course of an experiment that might affect the alignment of the channels. Channel alignment was performed before each imaging session using 100-nm TetraSpeck beads (T-7279, Invitrogen) and the ONI software aiming for an alignment error of SD < 8 nm as measured from 2000 points total across a maximum of 20 fields of view. Imaging was performed in near-TIRF (angle: 53.5°) using a motorized mirror and all images were acquired at 50 Hz.

Coverslips with neurons labeled as described above were mounted on concave slides in dSTORM-buffer (50 mM Tris, 10 mM NaCl, pH 8.0, supplemented with 5–20 mM MEA, 10% [w/v] glucose, 700 μg/ml glucose oxidase, and 40 μg/ml catalase). Transfected neurons were localized using low laser powers. At low laser powers, a snapshot was obtained of all channels, used later for selection of ROIs.

Dual-color dSTORM was performed simultaneously for CF568 and Alexa647. First, a pulse of high laser power of both the 561-nm and 640-nm lasers was used to bring the dyes into the dark state. Next, laser powers were lowered to around 100–200 mW. The acquisition was started when clear individual (non-overlapping) blinking was observed. 405-nm laser power was increased on demand based the number of blinking events. Imaging was continued for 20.000 frames or till no blinking events could be observed.

Localization coordinates were obtained and corrected for XY-drift using the ONI-software (version 1.12, software integrated with ONI microscope). From here onwards, analyses was continued in MATLAB (version 2019b). Localizations with a x–y-precision of > 50 nm were filtered out. Next, tracking with a tracking radius of 60 nm was performed to find localizations that were detected in multiple consecutive imaging frames. For each track, we selected the localization with the smallest localization error and filtered those further using a < 25 nm cutoff, being the final filtered dataset. Rendered reconstructions were made in Detection of Molecules (DoM), downloaded from: https://github.com/ekatrukha/DoM_Utrecht. Localization plots were made in MATLAB.

### Analysis of co-localization in microtubule dataset

Single-color dSTORM datasets were filtered on localization precision and tracking as described above. Next, the datasets were split by generating *n* unique random numbers between 1 and *n* using the MATLAB function *randperm*, were *n* is the number of localizations in the dataset. These numbers were used to assign localizations between two channels in ratios 1:1, 1:9 and 1:99. Translation was introduced by adding various distances to the x and y localizations of channel B. For rotations, the localizations of channel B were rotated around the center of the selected ROI by varying angles between 2 and 180 degrees. For the comparison with ClusDoC, we made use of the MATLAB function ClusDoC^32^, downloaded from: https://github.com/PRNicovich/ClusDoC. We used the default settings, i.e., 0.4 as co-localization cutoff. Data was plotted as the fraction of co-localizing localizations. For Coloc-Tesseler, we made use of the stand-alone software downloaded from: https://github.com/flevet/Coloc-Tesseler. For both comparisons, we used the data shown in (Fig. 3g,h), to make the comparison.

### Analysis of co-localization in neuronal synapses

For synaptic structures, we selected ROIs of 702 × 702 nm (6 × 6 pixels at a pixel size of 117 nm) which we subsequently analyzed for co-localization analysis as described above.

### Statistics

Statistical significance was tested with a Students *t*-test when comparing two groups. A *P* value below 0.05 was considered significant. If multiple groups were compared, statistical significance was tested with a one-way ANOVA followed by a Bonferroni’s multiple comparison test. In all figures, * was used to indicate a *P* value < 0.05, ** for *P* < 0.01, and *** for *P* < 0.001. Reported n is number of cells or ROIs as indicated in the text or figure legend, and all biological experiments were replicated in cultures from at least two independent preparations. Statistical analysis and graphs were prepared in GraphPad Prism, and figures were generated in Adobe Illustrator CC.

## Supplementary Information

Supplementary Information 1.

Supplementary Information 2.

## Data Availability

All data generated or analyzed during this study are included in this published article (and its Supplementary Information files). A test dataset (based on Fig. 3e,f) and scripts, together with a brief user guide are included as Supplementary information.
