# Supplementary material for: A coordinate-based co-localization index to quantify and visualize spatial associations in single-molecule localization microscopy
Source: Sci Rep. 2022 Mar 18;12:4676. doi: 10.1038/s41598-022-08746-4 (PMC8933590; doi:10.1038/s41598-022-08746-4)
Supplement: Supplementary file 1 — Supplementary Information 1. [file 41598_2022_8746_MOESM1_ESM.zip › Willems_at_al_supplementary_test_data/3th party scripts/crameri_v1.08/crameri/html/crameri_documentation.html]

crameri documentation 

# crameri documentation

crameri returns perceptually-uniform scientific colormaps created by Fabio Crameri.

## Contents

- Syntax
- Description
- Examples
- Citing this colormap:
- Author Info

## Syntax

```
crameri
cmap = crameri('ColormapName')
cmap = crameri('-ColormapName')
cmap = crameri(...,NLevels)
cmap = crameri(...,'pivot',PivotValue)
crameri(...)
```

## Description

crameri without any inputs displays the options for colormaps.

cmap = crameri('ColormapName') returns a 256x3 colormap. For a visual depiction of valid colormap names, type crameri.

cmap = crameri('-ColormapName') a minus sign preceeding any ColormapName flips the order of the colormap.

cmap = crameri(...,NLevels) specifies a number of levels in the colormap. Default value is 256.

cmap = crameri(...,'pivot',PivotValue) centers a diverging colormap such that white corresponds to a given value and maximum extents are set using current caxis limits. If no PivotValue is set, 0 is assumed.

crameri(...) without any outputs sets the current colormap to the current axes.

## Examples

Using this sample plot:

```
imagesc(peaks(1000)+1)
colorbar
```

Set the colormap to 'devon':

```
crameri devon
```

Same as above, but with an inverted devon colormap:

```
crameri -devon
```

Set the colormap to a 12-level 'hawaii':

```
crameri('hawaii',12)
```

Get the RGB values of a 5-level buda colormap:

```
RGB = crameri('buda',5)
```

```
RGB =
    0.7002    0.0027    0.7006
    0.7353    0.3060    0.5652
    0.8050    0.5211    0.4955
    0.8605    0.7409    0.4401
    1.0000    1.0000    0.4002
```

Some of the values in the image are below zero and others are above. If this dataset represents anomalies, perhaps a diverging colormap is more appropriate:

```
crameri('cork')
```

It's unlikely that 1.7776 is an interesting value about which the data values diverge. If you want to center the colormap on zero you can either change the color axis limits using caxis, or you can keep the current color axis limits by simply including the 'pivot' option when you call crameri:

```
crameri('cork','pivot',0)
```

For a final example, topography. Topography is a special case because it's usually important to clearly distinguish between land and ocean, but we still want a linear relationship between the perceived color and elevation. A typical divergent colormap like cork or vik might draw the eye toward the general direction of coastlines, but would not provide a clear definition between land and ocean, so the crameri colormaps include 'oleron', which is designed specifically for topography. Below I'm using my shadem function to apply relief shading.

```
load topo

figure
imagesc(topolonlim,topolatlim,topo)
axis xy
cb = colorbar;
ylabel(cb,'elevation')

shadem(-11,[225 83])
crameri('oleron','pivot')
```

## Citing this colormap:

Please acknowledge the free use of these colormaps by citing

Crameri, F. (2018). Scientific colour-maps. Zenodo. http://doi.org/10.5281/zenodo.1243862

Crameri, F. (2018), Geodynamic diagnostics, scientific visualisation and StagLab 3.0, Geosci. Model Dev., 11, 2541-2562, doi:10.5194/gmd-11-2541-2018.

For more on choosing effective and accurate colormaps for science, be sure to enjoy this fine beach reading:

Thyng, K.M., C.A. Greene, R.D. Hetland, H.M. Zimmerle, and S.F. DiMarco. 2016. True colors of oceanography: Guidelines for effective and accurate colormap selection. Oceanography 29(3):9-13, http://dx.doi.org/10.5670/oceanog.2016.66.

## Author Info

This function and supportind documentation were written by Chad A. Greene of the University of Texas Institute for Geophysics (UTIG), August 2018, using Fabio Crameri's scientific colormaps, version 7.0. http://www.fabiocrameri.ch/colourmaps.php

Published with MATLAB® R2020b
