# Supplementary figures and images for: A coordinate-based co-localization index to quantify and visualize spatial associations in single-molecule localization microscopy
Source: Sci Rep. 2022 Mar 18;12:4676. doi: 10.1038/s41598-022-08746-4 (PMC8933590; doi:10.1038/s41598-022-08746-4)

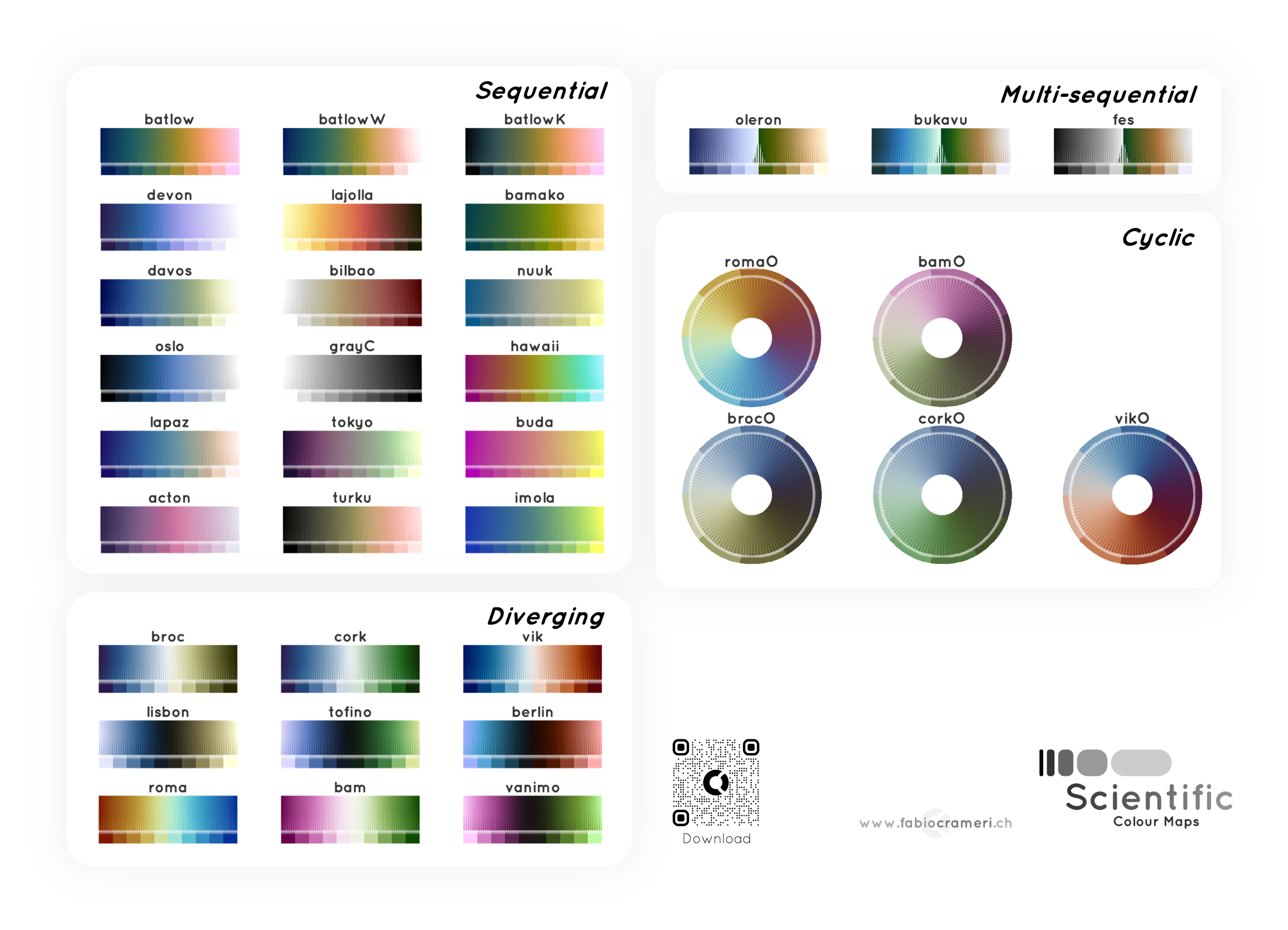

Supplement: Supplementary file 1 — Supplementary Information 1. [file 41598_2022_8746_MOESM1_ESM.zip › Willems_at_al_supplementary_test_data/3th party scripts/crameri_v1.08/crameri/crameri7.0.png]

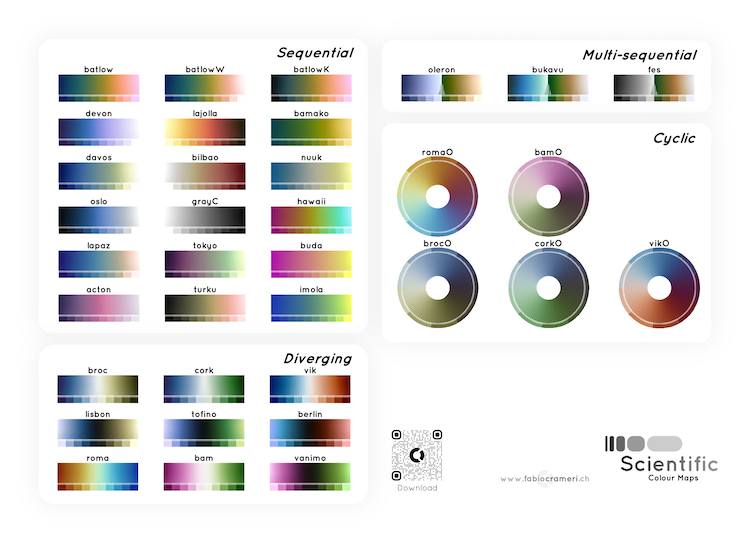

Supplement: Supplementary file 1 — Supplementary Information 1. [file 41598_2022_8746_MOESM1_ESM.zip › Willems_at_al_supplementary_test_data/3th party scripts/crameri_v1.08/crameri/html/crameri7.0_reduced.png]

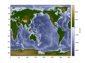

Supplement: Supplementary file 1 — Supplementary Information 1. [file 41598_2022_8746_MOESM1_ESM.zip › Willems_at_al_supplementary_test_data/3th party scripts/crameri_v1.08/crameri/html/crameri_documentation.png]

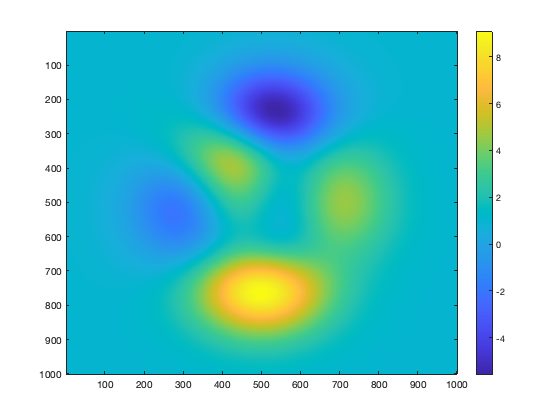

Supplement: Supplementary file 1 — Supplementary Information 1. [file 41598_2022_8746_MOESM1_ESM.zip › Willems_at_al_supplementary_test_data/3th party scripts/crameri_v1.08/crameri/html/crameri_documentation_01.png]

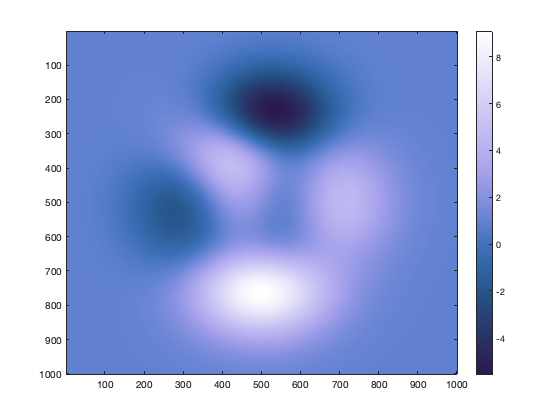

Supplement: Supplementary file 1 — Supplementary Information 1. [file 41598_2022_8746_MOESM1_ESM.zip › Willems_at_al_supplementary_test_data/3th party scripts/crameri_v1.08/crameri/html/crameri_documentation_02.png]

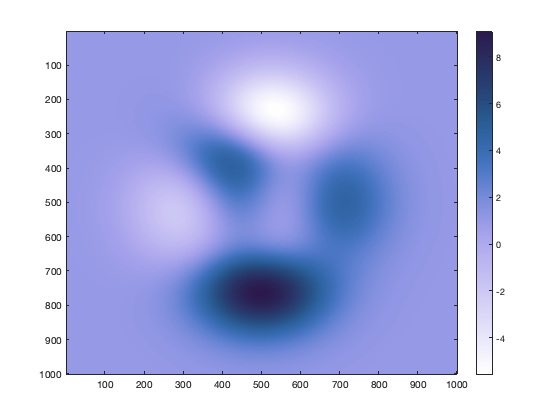

Supplement: Supplementary file 1 — Supplementary Information 1. [file 41598_2022_8746_MOESM1_ESM.zip › Willems_at_al_supplementary_test_data/3th party scripts/crameri_v1.08/crameri/html/crameri_documentation_03.png]

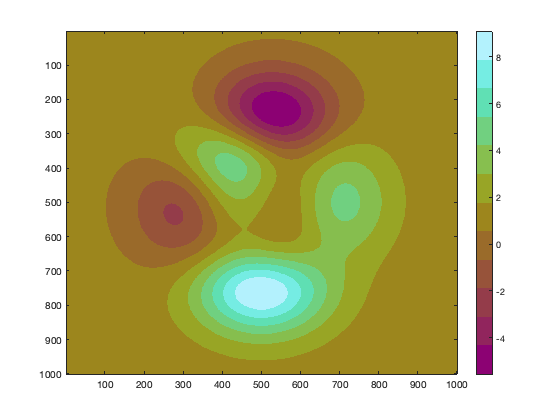

Supplement: Supplementary file 1 — Supplementary Information 1. [file 41598_2022_8746_MOESM1_ESM.zip › Willems_at_al_supplementary_test_data/3th party scripts/crameri_v1.08/crameri/html/crameri_documentation_04.png]

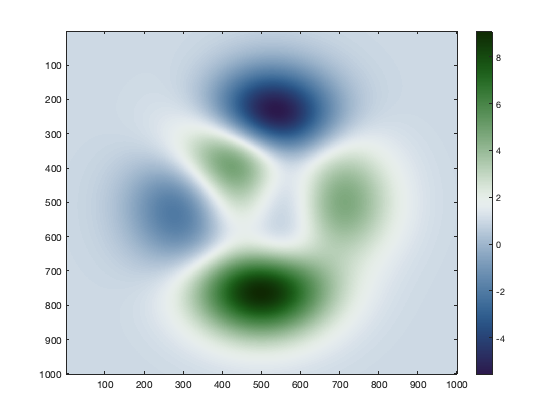

Supplement: Supplementary file 1 — Supplementary Information 1. [file 41598_2022_8746_MOESM1_ESM.zip › Willems_at_al_supplementary_test_data/3th party scripts/crameri_v1.08/crameri/html/crameri_documentation_05.png]

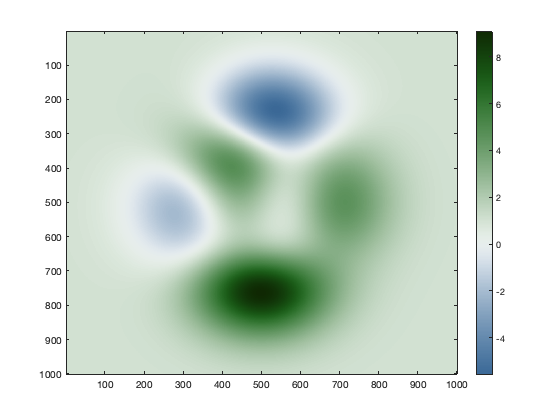

Supplement: Supplementary file 1 — Supplementary Information 1. [file 41598_2022_8746_MOESM1_ESM.zip › Willems_at_al_supplementary_test_data/3th party scripts/crameri_v1.08/crameri/html/crameri_documentation_06.png]

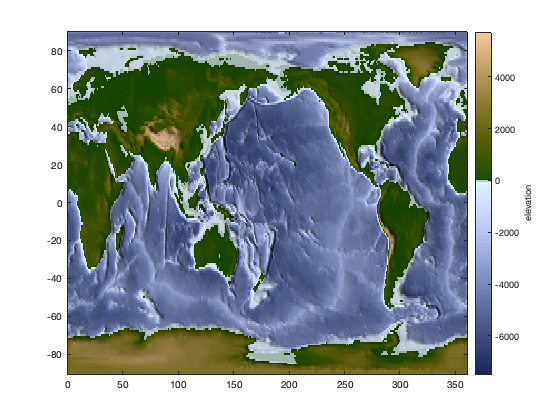

Supplement: Supplementary file 1 — Supplementary Information 1. [file 41598_2022_8746_MOESM1_ESM.zip › Willems_at_al_supplementary_test_data/3th party scripts/crameri_v1.08/crameri/html/crameri_documentation_07.png]
