# Supplementary material for: A coordinate-based co-localization index to quantify and visualize spatial associations in single-molecule localization microscopy
Source: Sci Rep. 2022 Mar 18;12:4676. doi: 10.1038/s41598-022-08746-4 (PMC8933590; doi:10.1038/s41598-022-08746-4)
Supplement: Supplementary file 2 — Supplementary Information 2. [file 41598_2022_8746_MOESM2_ESM.pdf]

A coordinate-based co-localization index to quantify and visualize spatial associations in single-molecule localization microscopy

Jelmer Willems and Harold D. MacGillavry*

Division of Cell Biology, Neurobiology and Biophysics, Department of Biology, Faculty of Science, Utrecht University, 3584 CH, Utrecht, The Netherlands.

*Correspondence to: h.d.macgillavry@uu.nl

MATLAB Code S1

```
function CIX = CI_index(x1, y1, x2, y2,LP1,LP2)
% ; NAME:
% ;                  CI_index
% ; PURPOSE:
% ;                  Calculates the co-localization index between two channels
in SMLM datasets
% ;
% ; CALLING SEQUENCE:
% ;                  CIX = CI_index(x1, y1, x2, y2, LP1, LP2)
% ;
% ; INPUTS:
% ;                  x1 and y1 being the coordinates of the first channel
% ;                  x2 and y2 being the coordinates of the second channel
% :                  LP1 and LP2 being the average localization precision of x
and y (in the same units as x and y)
% ;
% ; OUTPUTS:
% ;                  CIX:    struct with a row for each channel containing the
fields:
% ;                  .NND_each_loc          : containing the nearest neighbor of
each localization
% ;                  .MNND                  : mean nearest neighbor distance
% ;                  .effective_resolution : effective resolution used as search
radius
% ;                  .LD_each_loc           : local-density value of all
localizations
% ;                  .MLD                   : local-density value averaged over
all localizations
% ;                  .opposing_LD_each_loc : local-density each localization
experiences in the other channel
% ;                  .CI_each_loc           : Co-localization index of each
localization
% ;                  .mean_CI               : Co-localization index averaged over
all localizations
% ;
% ;
% ; Jelmer Willems, 2021
% ;
% ;

        % LOCAL-DENSITY
        channel=1;
        while channel <3
              if channel == 1
                 x = x1;
                 y = y1;
                 LP = LP1;
              else
                 x = x2;
                 y = y2;
                 LP = LP2;
              end

              % find NND distance
```

```
            [IDX,D]=knnsearch([x,y],[x,y],'k',2);
            MNND=mean(D(:,2));

            % save NND and MNND in output struct
            CIX(channel).NND_each_loc=D(:,2);
            CIX(channel).MNND=MNND;

            % determine effective resolution as search radius
            effective_resolution=sqrt((MNND^2)+(LP^2));
            CIX(channel).effective_resolution=effective_resolution;

            % find all localizations in search radius
            IDR=rangesearch([x,y],[x,y],effective_resolution);

            % determine local density for each localization and the mean of
all loc
            local_density = zeros(1,length(IDR))';
            for alllocs = 1:length(IDR)
                local_density(alllocs,1) = length(IDR{alllocs,1});
            end
            mean_local_density = mean(local_density);

            % save local density in output struct
            CIX(channel).LD_each_loc = local_density;
            CIX(channel).MLD = mean_local_density;

            channel=channel+1;
        end

        % CO-LOCALIZATION INDEX
        active_channel = 1;
        while active_channel <3

            % selection of required data for active channel
            if active_channel == 1
               localizations_assayX = x1;
               localizations_assayY = y1;
               localizations_otherX = x2;
               localizations_otherY = y2;
               other_channel = 2;
            else
               localizations_assayX = x2;
               localizations_assayY = y2;
               localizations_otherX = x1;
               localizations_otherY = y1;
               other_channel = 1;
            end
            mean_local_density_other = CIX(other_channel).MLD;
            effective_resolution = CIX(other_channel).effective_resolution;

            % find all neighbors in the opposing channel within the search
radius

IDQ=rangesearch([localizations_otherX,localizations_otherY],[localizations_as
sayX,localizations_assayY],effective_resolution);
```

```
            % determine local density of all localizations in opposing
channel
            opposing_local_density = zeros(1,length(IDQ))';
            for alllocs=1:length(IDQ)
                opposing_local_density(alllocs,1)=length(IDQ{alllocs,1});
            end

            % normalize to mean local density to obtain co-localization
index

CI_index_all_localization=opposing_local_density/(mean_local_density_other-
1);
            Mean_CI_ROI=mean(CI_index_all_localization);

            % save in output struct

CIX(active_channel).opposing_LD_each_loc=opposing_local_density;
            CIX(active_channel).CI_each_loc=CI_index_all_localization;
            CIX(active_channel).Mean_CI=Mean_CI_ROI;

            active_channel=active_channel+1;
        end
end
```

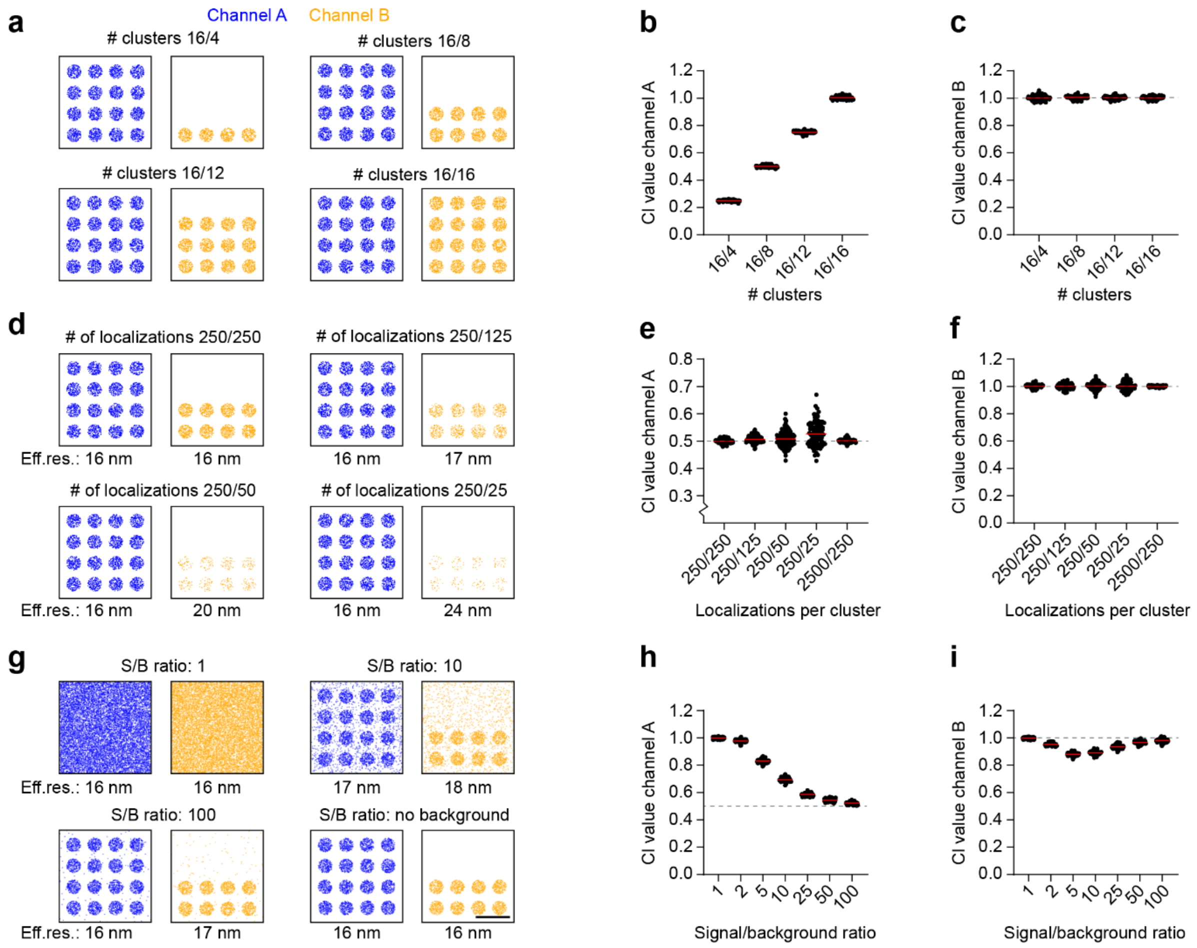

Supplementary Figure S1, related to figure 1: Co-localization measurements on multiple clusters with varying densities and background

(a) Simulations of multiple clusters in a channel, varying the number of clusters plotted in the second channel. **(d)** Simulations showing 16 clusters in channel A and 8 clusters in channel B, titrating the density of the clusters in channel B. **(g)** Simulations similar to (a,d) but with changing signal/background density ratios. Scale bar, 500 nm. **(b,e,h)** Co-localization in channel A as measured for the varius conditions of which some are shown in (a,d,g). **(c,f,i)** Co-localization values for channel B. In all graphs, the dotted line indicates the theoretical overlap between the clusters and without background. Average effective resolution is shown below the conditions. CI, co-localization index; Eff. Res, effective resolution; S/B, Signal/background.

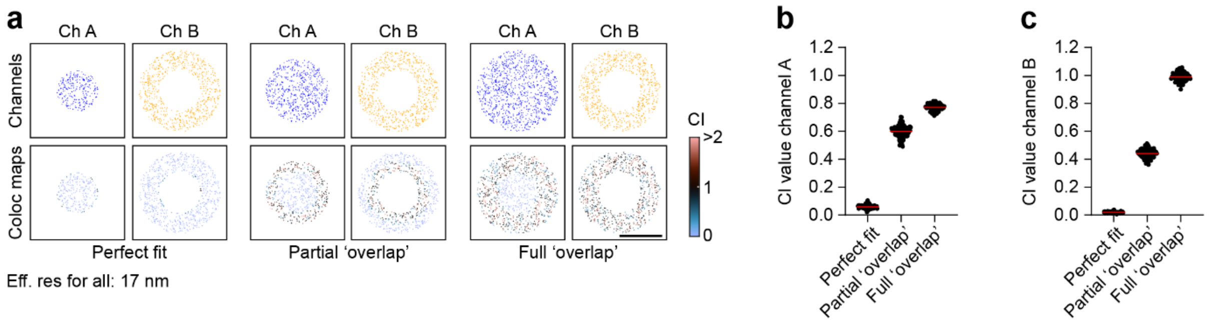

Supplementary Figure S2, related to figure 1: Co-localization of surrounding spatial distribution

(a) Simulated clusters in which channel B (yellow) is surrounding channel A (blue), with no overlap (perfect fit), partial overall and full overlap (albeit no overlap in the center). Co-localization maps show that co-localization is only measured in those areas were co-localization occurs. Scale bar, 250 nm. **(b,c)** Graphs showing co-localization values measured for channel A and B respectively across the conditions shown in (a). CI, co-localization index; Eff. Res, effective resolution; Ch, channel.

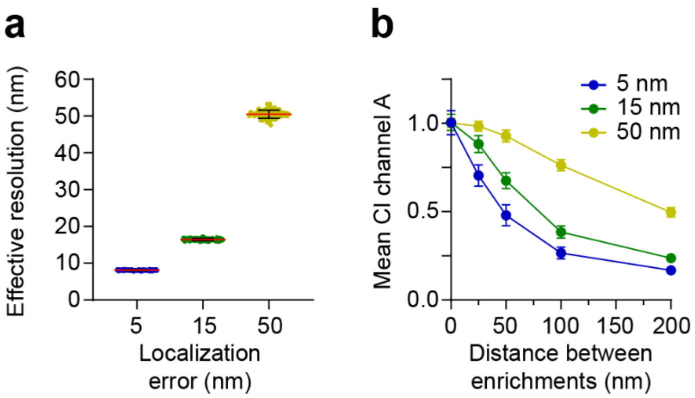

Supplementary Figure S3, related to figure 2E: Effects of changing effective resolution on measuring co-localization

(a) Effective resolution over the varying localization errors but constant localization densities. **(b)** Graphs showing mean co-localization index measured at clusters decreasing overlap at three different localization errors (5 nm, 15 nm and 50 nm). Data are represented as means ± SEM. ROI, region of interest.

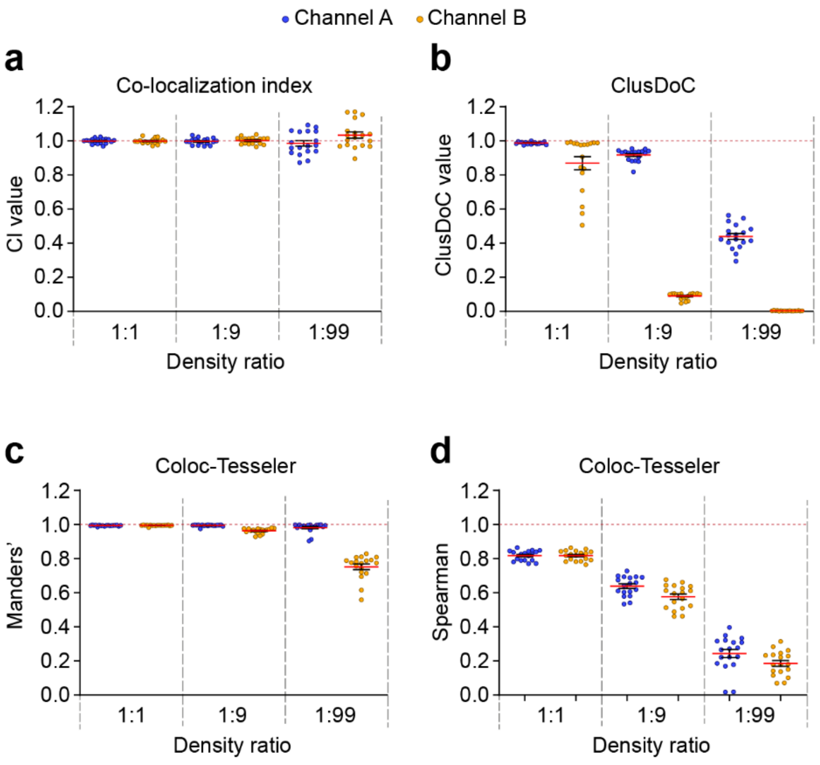

Supplementary Figure S4, related to figure 3g,h: Comparison of co-localization index with ClusDoC and Coloc-Tesseler

(a) Replicate of graph show as figure 3h in the mean text (for comparison), showing the co-localization index as measured across ROIs with different relative localization densities. **(b)** Co-localization as measured with ClusDoC. Values show percentage of co-localizing localizations (as fraction) across the density ratios. **(c,d)** Coloc-Tesseler analysis of co-localization, with both Manders' (c) and Spearman correlation (d) output measurements across the measured density ratios. Orange dotted line indicates the theoretical co-localization between the channels.
